# Supplementary material for: Elevated Hepcidin Expression in Human Carotid Atheroma: Sex-Specific Differences and Associations with Plaque Vulnerability
Source: Int J Mol Sci. 2024 Jan 30;25(3):1706. doi: 10.3390/ijms25031706 (PMC10855936; doi:10.3390/ijms25031706)
Supplement: Supplementary file 1 [file ijms-25-01706-s001.zip › ijms-2803831-supplementary.pdf]

Supplementary Table SI. Basic clinical information

	Total	Asymptomatic	Symptomatic	
n	58	8	50	p
Age, y ± SE	71.7 ± 1.1	74.4 ± 2.2	71.2 ± 1.2	ns
Sex, male (%, n)	65.5 (38)	62.5 (5)	66.0 (33)	ns
Diabetes mellitus, % (n)	17.2 (10)	25.0 (2)	16.0 (8)	ns
Hypertesion, % (n)	74.1 (43)	62.5 (5)	76.0 (38)	ns
Smoking, % (n)	41.4 (24)	25.0 (2)	44.0 (22)	ns
Statin treatment, % (n)	46.5 (27)	62.5 (5)	44.0 (22)	ns
Total cholesterol	5.1 ± 0.2	5.1 ± 0.4	5.1 ± 0.2	ns
LDL	2.8 ± 0.2	2.5 ± 0.5	2.8 ± 0.2	ns
HDL	1.3 ± 0.1	1.9 ± 0.4	1.2 ± 0.1	<0.05
TG	2.5 ± 0.4	1.6 ± 0.3	2.7 ± 0.5	ns
